# Efficacy and safety of the traditional Chinese medicine tonifying kidney (bu shen) therapy in patients with hypertension: A protocol for systematic review and meta-analysis

**DOI:** 10.1097/MD.0000000000021144

**Published:** 2020-07-17

**Authors:** Zhuoran Tang, Yize Sun, Chao Wang, Xiang Liu, Xin Qi, Di Ma, Haibin Zhao

**Affiliations:** a Beijing University of Chinese Medicine; b Dongfang Hospital; c The Third Affiliated Hospital, Beijing University of Chinese Medicine, Beijing, China.

**Keywords:** hypertension, protocol, systematic review, tonifying kidney

## Abstract

**Background:**

Hypertension becomes increasingly an alarming global health concern. There is a growing interest in treatment of traditional Chinese medicine (TCM), and tonifying kidney therapy (bushen, TKT) has been extensively used in the treatment of hypertension according to TCM theory. In this article, we outline the protocol of research projects and methods to examine comprehensively the effectiveness and safety of TKT in treating hypertensive patients.

**Methods:**

We will collect randomized controlled trails (RCTs) that report the application of TKT for patients with hypertension from electronic databases including PubMed, EMBASE, CENTRAL, CNKI, VIP, CBM, and Wanfang database. Time of literature retrieval is set from the beginning of database construction to the end of June, 2020. Two reviewers will independently perform literature screening, data extraction, and quality assessment of included literature, and any divergences will be worked out via discussion. The primary outcomes include total efficacy rate, systolic and diastolic blood pressure change will be assessed. The secondary outcomes include clinical symptoms and adverse events will also be assessed. RevMan5.3 software will be applied to analyzing data included studies.

**Results:**

This study will synthesize and analyze all collated data in order to evaluate TKT for the treatment of hypertension involves different aspects in total efficacy rate, systolic and diastolic blood pressure change, clinical symptoms, and adverse events.

**Conclusion:**

This study will determine the efficacy of TKT in the treatment of hypertension and recommend its clinical value based on the evaluated the effectiveness and security results.

**Registration number:**

INPLASY202050044.

## Abstract 2

Supplemental Digital Content is available in the text

## 1 Introduction

Hypertension is currently defined as consistent elevated blood and the patients diagnosed with the disease have systolic blood pressure ≥140 mm Hg, and/or diastolic blood pressure ≥90 mm Hg.^[1,2]^ Hypertension becomes increasingly an alarming global healthcare concern, which ultimately leads to heart disease, stroke, and renal insufficiency and premature mortality and disability,^[3]^ with almost three-fourths of the population with hypertension living in low-income and middle-income countries.^[4]^ According to the latest Chinese hypertension screening survey, the prevalence of hypertension was 27.9% and the estimated number of adult hypertensive patients is 244.5 million, besides, another estimated 435.3 million with pre-hypertension.^[5,6]^ In 2010, hypertension caused 9.4 million deaths, the number of adults diagnosed with hypertension was expected to rise to ∼1.56 billion by 2025.^[7,8]^ Furthermore, with the environmental changes, ageing, unhealthy lifestyle such as excessive sodium consumption, insufficient intake of dietary potassium, smoking, drinking, unhealthy diet, overweight and obesity, and physical inactivity constantly spring up,^[9,10]^ hypertension will continue to be an formidable challenge for the global healthcare industry. Hypertensive patients ought to consistently take conventional antihypertensive drugs, whereas those antihypertensive drugs are likely to develop certain side effects. Globally, the application of CAM to manage cardiovascular disease is on the rise.^[11,12]^ And among patients who use CAM, the most common forms are antihypertensive herbal medicines.^[11,13]^ Many previous studies have revealed that Chinese herbal medicines (e.g., Tianma Gouteng Decoction, Jian Ling Decoction, Xiao Yao San) associated with conventional therapy is further effective and safer for treating hypertension than conventional therapy only.^[14–17]^

According to TCM theory, hypertension coincides with the category of “vertigo” or “headache” on the basis of its clinical manifestations, which is originally documented in *Inner Canon of Yellow Emperor (also called Huangdi Neijing).* Kidney (shen) deficiency is one of the main mechanisms of hypertension,^[18,19]^ especially for the elderly and long duration of hypertension. Kidney deficiency syndrome involves Yin deficiency syndrome and Yang deficiency syndrome. Owing to chronic diseases can involve the kidney, kidney deficiency syndrome is always associated with high blood pressure.^[20]^ Many systematic reviews has confirmed that Chinese medicine prescriptions representing the kidney-tonifying method, such as Liuwei Dihuang pill, Qiju Dihuang pill, and Shenqi pill,^[21–23]^ have remarkable curative effects as an adjunctive therapy for reducing hypertension.

Although 1 systematic review on treating senile hypertensive patients with kidney-tonifying Chinese herbal formula was published 6 years ago, there have been many randomized controlled clinical studies published recently. In addition, further research is warranted to investigate the curative effect of hypertension with TKT regardless of age, sex or ethnic background. Therefore, we intend to perform this systematic review to update the clinical evidence of TKT in the treatment of hypertension based on its effectiveness and safety.

## 2 Methods

### 2.1 Protocol registration

The study protocol was registered with INPLASY (registration number: INPLASY202050044). The procedure of this protocol will be conducted in compliance with guidelines of PRISMA-P.^[24]^ If there are any adjustments of subtle details in this study, we intend to modify and update in the final publication.

### 2.2 Inclusion criteria

#### 2.2.1 Type of studies

We only collect RCTs that report the application of TKT for patients with hypertension, irrespective of whether using blind method or not.

#### 2.2.2 Type of participants

Participants included must be diagnosed with hypertension, whether the diagnostic criteria were described in literature. There will be no restrictions on age, sex, or ethnic background.

#### 2.2.3 Intervention measures

Intervention measures included TKT alone or combined with conventional western medication in the experiment group, whereas the control group adopted conventional western medication only. Furthermore, we will not set limitations on dosages, formulation composition and course of treatment.

#### 2.2.4 Outcome indicators

The primary outcome indicators involve total efficacy rate, systolic and diastolic blood pressure change. Clinical symptoms and adverse events will be ragarded as the secondary outcome indicators.

### 2.3 Literature retrieval

#### 2.3.1 Electronic searches

English online databases mainly include PubMed, EMBASE, CENTRAL; Chinese online databases mainly include CNKI, VIP, CBM, and Wanfang databases. Time of literature retrieval is set from the beginning of those database constructions to the end of June 2020. Literature must be published in English or Chinese. Finally, the search strategy of each database will be checked and modified according to our requirement. The retrieval strategy for PubMed will be presented as an example in Appendix A (Supplemental Appendix A), and other databases will be referenced to the retrieval strategy of PubMed.

#### 2.3.2 Searching other resources

Grey literature and clinical trial registries will be retrieved to compensate for the deficiencies of electronic databases.

### 2.4 Data extraction and synthesis

#### 2.4.1 Data extraction process

**Figure 1:**
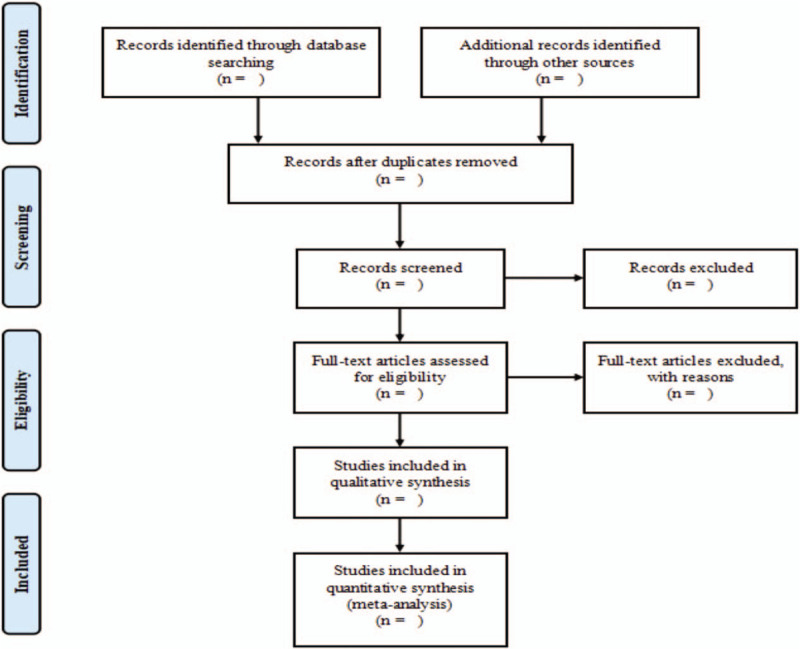
Flow diagram of study selection.

To import all literature into NoteExpress v3.2.0 software. Two reviewers (Zhuoran Tang and Yize Sun) will independently screen literature and conduct data extraction with pre-designed data extraction table and extraction results will be cross-checked by each other. Data extraction based on the following items: title, first author, publication year, diagnosis criteria, demographic information, study characteristics, sample size, interventions, outcomes, adverse events, etc. Divergences will be worked out via discussion with a third reviewer (Haibin Zhao). If relevant data is incomplete, we intend to contact the author of the original literature by telephone or email. We will analyze only available data of included literature when missing data cannot be obtained. PRISMA flow diagram will be applied to present the procedure of studies selection (Fig. 1). Moreover, all collated data will be imported into RevMan5.3 software.

#### 2.4.2 Assessment of bias

Two reviewers (Zhuoran Tang and Xiang Liu) will utilize RoB2 tool provided by the Cochrane Handbook version 6.0^[25]^ to evaluate bias risk. The main details of assessment include: random sequence generation, allocation concealment, types of blinding, missing outcome data, and other bias. The overall risk-of-bias evaluation is divided into three criteria: “low,” “high,” and “unclear ” bias. Disagreements will be reached consensus via discussion.

#### 2.4.3 Data synthesis

RevMan5.3 software will be used to integrate and analyze included studies. Dichotomous data will be reported as risk ratio (RR) with 95% confidence interval (CI), whereas continuous data will be reported as mean difference (MD) or standard mean difference (SMD) with 95% CI. Results of the meta-analysis will be visualised by forest plots.

#### 2.4.4 Assessment of heterogeneity

Heterogeneity is influenced by many factors (e.g., age, different intervention forms, missing data, etc), and we plan to evaluate its degree through *χ*^*2*^ test and *I*^2^test.

If *I*^2^ statistic <50% and *P* value > .1, we will consider that there exists statistical heterogeneity can be ignored and adopt the fixed model to integrate the data. Conversely, the random effect model will be adopted when *I*^*2*^statistic ≥50% and *P* value < .1. Furthermore, we will conduct subgroup analysis or sensitivity analysis to investigate potential sources of heterogeneity.

#### 2.4.5 Publication bias

Funnel plots will be applied to examine publication bias when at least 10 trials are identified.

#### 2.4.6 Subgroup analysis

If sufficient comparable studies are available, subgroup analysis will be conducted in terms of age, sex, intervention forms, treatment course, etc.

#### 2.4.7 Sensitivity analysis

In order to ensure the robustness of analytical conclusions, we will utilize sensitivity analysis to examine the impact of low quality trials.

#### 2.4.8 Ethnics and dissemination

It requires no ethical approval for this study on the basis of collecting and collating documents. Findings will be disseminated through a peer-reviewed publication.

#### 2.4.9 Evidence assessment

Conforming to the GRADE approach, we evaluate the strength of evidence for each outcome with rated as “high,” “moderate,” “low,’, and “very low.”

## 3 Discussion

In recent years, more and more people attach importance to TCM therapy, which has been proved to have a conspicuousness impact on blood pressure control in many previous clinical trials of TCM for hypertension. Animal experiments on TCM to treat hypertension are also being explored. Wang et al^[26]^ found that Qin-Dan-Jiang-Ya-Tang (QDJYT) might be associated with inhibiting bFGFmRNA and its proteic productions to effectively lower blood pressure and improve vascular remodeling in spontaneous hypertensive rats. Xiong et al^[27]^ suggested that Bu-Shen-Jiang-Ya decoction (BSJYD) combined with conventional western medicine could effectively manage blood pressure and heart rate in order to reverse ventricular remodeling of spontaneous hypertensive rats via the mechanism that BSJYD may suppress EKR signaling pathway. In China, it is widely known among TCM clinicians that “kidney”(Shenzang) is vital to treat hypertensive patients, especially for patients diagnosed as “kidney deficiency” after based on syndrome differentiation of TCM need to adopt TKT. Hypertensive patients with “kidney deficiency” syndrome (also called “zheng” or “pattern”) such as nocturia, fatigue, vertigo, tinnitus, insomnia, hot flashes, waist soreness, and knee weakness could take Qiju Dihuang capsule or Jinkui Shenqi pill based on the difference of “ kidney yin deficiency” or “kidney yang deficiency”.^[28]^ Among antihypertensive drugs, diuretics and beta-blockers may lead to harmful impacts on sexual function.^[29]^ Besides, antihypertensive drugs can also lead to insomnia, fatigue, joint pain, dizziness, tinnitus, cold hands and feet. According to TCM theory, “kidney govern reproduction” and “kidney govern bone” illustrate that kidney is the basis of life, however hypertensive patients are usually accompanied by kidney damage. TKT in treating hypertensive patients based on diverse clinical manifestations, including tonifying kidney only, tonifying kidney and activating blood (Huoxue), tonifying kidney and soothing liver (Shugan) during clinical practice.

With the emergence of many clinical trials on TKT for hypertension recently, we hope to provide higher evidence than the previous meta-analysis through analyzing the latest trials regarding this topic and expanding the sample size. However, there might be some deficiencies in this study, which may lead to heterogeneity due to small sample size, age, dosage, intervention forms, and treatment course.

## Author contributions

**Conceptualization:** Zhuoran Tang.

**Data curation:** Chao Wang, Haibin Zhao.

**Formal analysis:** Zhuoran Tang.

**Funding acquisition:** Haibin Zhao, Di Ma.

**Investigation:** Zhuoran Tang, Chao Wang.

**Methodology:** Zhuoran Tang, Haibin Zhao.

**Project administration:** Haibin Zhao.

**Resources:** Zhuoran Tang, Yize Sun, Xiang Liu.

**Software:** Xiang Liu, Xin Qi, Di Ma.

**Supervision:** Haibin Zhao.

**Writing – original draft:** Zhuoran Tang, Yize Sun.

**Writing – review & editing:** Zhuoran Tang.

## Supplementary Material

Supplemental Digital Content

## References

[1] Liu J Highlights of the 2018 Chinese hypertension guidelines. Clin Hypertens 2020;26:8. 32377372 10.1186/s40885-020-00141-3 PMC7193361

[2] Mancia G Fagard R Narkiewicz K 2013 ESH/ESC guidelines for the management of arterial hypertension: the Task Force for the Management of Arterial Hypertension of the European Society of Hypertension (ESH) and of the European Society of Cardiology (ESC). Eur Heart J 2013;34:2159–219. 23771844 10.1093/eurheartj/eht151

[3] World Heath Organization. A global brief on hypertension: silent killer, global public health crisis. World Health Day;2013.

[4] Mills KT Bundy JD Kelly TN Global disparities of hypertension prevalence and control: a systematic analysis of population-based studies from 90 countries. Circulation 2016;134:441–50. 27502908 10.1161/CIRCULATIONAHA.115.018912 PMC4979614

[5] Guo QH Zhang YQ Wang JG Asian management of hypertension: current status, home blood pressure, and specific concerns in China. J Clin Hypertens (Greenwich) 2020;22:475–8. 31622005 10.1111/jch.13687 PMC8029819

[6] Wang Z Chen Z Zhang L Status of hypertension in China: results from the China Hypertension Survey, 2012–2015. Circulation 2018;137:2344–56. 29449338 10.1161/CIRCULATIONAHA.117.032380

[7] Kintscher U The burden of hypertension. Eurointervention 2013;9: Suppl R: R12–5. 23732143 10.4244/EIJV9SRA3

[8] Kearney PM Whelton M Reynolds K Global burden of hypertension: analysis of worldwide data. Lancet 2005;365:217–23. 15652604 10.1016/S0140-6736(05)17741-1

[9] Poulter NR Prabhakaran D Caulfield M Hypertension. Lancet 2015;386:801–12. 25832858 10.1016/S0140-6736(14)61468-9

[10] Cherfan M Vallee A Kab S Unhealthy behavior and risk of hypertension: the CONSTANCES population-based cohort. J Hypertens 2019;37:2180–9. 31584898 10.1097/HJH.0000000000002157

[11] Anwar MA Al DS Eid AH Anti-hypertensive herbs and their mechanisms of action: part II. Front Pharmacol 2016;7:50. 27014064 10.3389/fphar.2016.00050 PMC4782109

[12] Frass M Strassl RP Friehs H Use and acceptance of complementary and alternative medicine among the general population and medical personnel: a systematic review. Ochsner J 2012;12:45–56. 22438782 PMC3307506

[13] Osamor PE Owumi BE Complementary and alternative medicine in the management of hypertension in an urban Nigerian community. BMC Complement Altern Med 2010;10:36. 20642829 10.1186/1472-6882-10-36 PMC2912779

[14] Tai J Zou J Zhang X Randomized controlled trials of Tianma Gouteng Decoction combined with Nifedipine in the treatment of primary hypertension: a systematic review and meta-analysis. Evid Based Complement Alternat Med 2020;2020:5759083. 32089726 10.1155/2020/5759083 PMC7029275

[15] Xiong X Wang P Li X The effect of Chinese herbal medicine Jian Ling Decoction for the treatment of essential hypertension: a systematic review. Bmj Open 2015;5:e6502. 10.1136/bmjopen-2014-006502 PMC4322192 25652798

[16] Xiong X Wang P Duan L Efficacy and safety of Chinese herbal medicine Xiao Yao San in hypertension: a systematic review and meta-analysis. Phytomedicine 2019;61:152849. 31035044 10.1016/j.phymed.2019.152849

[17] Xinke Z Yingdong L Mingxia F Chinese herbal medicine for the treatment of primary hypertension: a methodology overview of systematic reviews. Syst Rev 2016;5:180. 27760557 10.1186/s13643-016-0353-y PMC5072301

[18] Wang J Xiong XJ Liu W Discussion on treatment of hypertension by tonifying kidney. Zhongguo Zhong Yao Za Zhi 2013;38:1277–9. 23944051

[19] Wang LY Shi NN Han XJ Study on TCM syndrome distribution of 1508 cases of hypertension patients with clinical epidemiology. China J Tradit Chin Med Pharm 2010;25:1960–3.

[20] Wang J Xiong X Control strategy on hypertension in Chinese medicine. Evid Based Complement Alternat Med 2012;2012:284847. 22194771 10.1155/2012/284847 PMC3239016

[21] Guo YX Chen XL Qiu ZW Meta-analysis and systematic review of Liuweidihuang pills combined with Western Medicine on Hypertension. J Emerg Tradit Chin Med 2013;22:189–91. +210.

[22] Ju JQ Li YL Yang CH Systematic review of clinical efficacy and safety of QijuDihuang pills in treating essential hypertension. J Shandong Univ Tradit Chin Med 2013;37:363–7.

[23] Xiong X Wang P Li X Shenqi pill, a traditional Chinese herbal formula, for the treatment of hypertension: a systematic review. Complement Ther Med 2015;23:484–93. 26051584 10.1016/j.ctim.2015.04.008

[24] Shamseer L Moher D Clarke M Preferred reporting items for systematic review and meta-analysis protocols (PRISMA-P) 2015: elaboration and explanation. BMJ 2015;350:g7647 doi:10.1136/bmj.g7647. 25555855 10.1136/bmj.g7647

[25] Higgins JPT, Thomas J, Chandler J, et al. Cochrane handbook for systematic reviews of interventions version 6.0 (updated July 2019). Cochrane; 2019. Available at www.training.cochrane.org/handbook. Accessed June 1, 2020.

[26] Wang B Zhang JD Feng JB Improvement of vascular remodeling in spontaneous hypertensive rats with traditional Chinese medicine. Clin Exp Hypertens 2007;29:345–55. 17653968 10.1080/10641960701500612

[27] Xiong X Yang X Duan L Traditional Chinese medicine suppresses left ventricular hypertrophy by targeting extracellular signal-regulated kinases signaling pathway in spontaneously hypertensive rats. Sci Rep 2017;7:42965 doi:10.1038/srep42965. 28225023 10.1038/srep42965 PMC5320505

[28] Wang LY Chan KW Yuwen Y Expert consensus on the treatment of hypertension with Chinese patent medicines. Evid Based Complement Alternat Med 2013;2013:510146. 23662141 10.1155/2013/510146 PMC3638588

[29] Imprialos KP Stavropoulos K Doumas M Sexual dysfunction, cardiovascular risk and effects of pharmacotherapy. Curr Vasc Pharmacol 2018;16:130–42. 28595561 10.2174/1570161115666170609101502

