# Supplementary material for: Efficacy and safety of the traditional Chinese medicine tonifying kidney (bu shen) therapy in patients with hypertension: A protocol for systematic review and meta-analysis
Source: Medicine (Baltimore). 2020 Jul 17;99(29):e21144. doi: 10.1097/MD.0000000000021144 (PMC7373579; doi:10.1097/MD.0000000000021144)
Supplement: Supplemental Digital Content [file medi-99-e21144-s001.doc]

Supplemental content

Appendix A.

Search strategy used in PubMed database

#1 **("Hypertension"[Mesh]) OR ((((((essential hypertension[Title/Abstract])) OR (primary hypertension[Title/Abstract])) OR (hypertension, malignant[Title/Abstract])) OR (resistant hypertension[Title/Abstract]))**

#2 (tonifying kidney) OR (bu shen) OR (nourishing kidney) OR (tonifying the kidney) OR (reinforcing the kidney) OR (zi shen) OR (wen shen) OR (warm the kidney)

#3 Randomized controlled trial OR clinical study OR Clin-ical Trial OR Controlled study OR Controlled Trial OR Random*Control* study OR random* Control* Trial

#4 #1 AND #2 AND #3
